# Chinese Medicine Formula PSORI-CM02 Alleviates Psoriatic Dermatitis via M-MDSCs and Th17 Crosstalk

**DOI:** 10.3389/fphar.2020.563433

**Published:** 2021-01-18

**Authors:** Jingwen Deng, Siyi Tan, Ruonan Liu, Wanlin Yu, Haiming Chen, Nan Tang, Ling Han, Chuanjian Lu

**Affiliations:** ^1^ The Second Affiliated Hospital of Guangzhou University of Chinese Medicine, Guangzhou, China; ^2^ Guangdong Provincial Key Laboratory of Clinical Research on Traditional Chinese Medicine Syndrome, Guangdong Provincial Hospital of Chinese Medicine, Guangzhou, China; ^3^ Guangzhou Panyu Central Hospital, Guangzhou, China; ^4^ Department of Physiology, Medical College, Jinan University, Guangzhou, China; ^5^ Central Laboratory, Guangdong Provincial Hospital of Chinese Medicine, Guangzhou, China; ^6^ Guangzhou Red Cross Hospital, Medical College, Jinan University, Guangzhou, China

**Keywords:** Chinese medicine, PSORI-CM02, Th17, MDSCs, psoriasis

## Abstract

Psoriasis is a chronic inflammatory skin disease that is associated with multiple coexisting conditions. Extensive literature suggests that psoriasis is a T-cell-mediated condition, and its pathogenesis is related to dysfunction of the immune system. Myeloid-derived suppressor cells (MDSCs) are a group of heterogeneous myeloid cells that have suppressive effects on T cells. MDSCs are present at very low levels in healthy individuals but can substantially expand in tumours or inflammatory conditions. PSORI-CM02, a Chinese medical formula designed based on the Chinese medicine theory (*Blood Stasis*), has been prescribed extensively for psoriasis therapy and shows a stable clinical effect and safety. This study discusses the mechanisms of MDSCs involved in disease development and therapeutic progress. Our data provides evidence that monocytic myeloid-derived suppressor cells (M-MDSCs) play a role in IMQ-induced psoriatic dermatitis. Functional characterization and correlation analysis indicated that MDSCs are positively correlated with Th17 cells. PSORI-CM02 alleviated IMQ-induced psoriatic dermatitis and suppressed the proliferation of Th17 cells via M-MDSC-induced Arg1 upregulation, suggesting M-MDSCs could be a novel therapeutic target for psoriasis, and PSORI-CM02 exerted its effects via the perturbation of M-MDSCs and Th17 cell crosstalk.

## Introduction

Psoriasis is a common skin disease caused by both autoimmune dysfunction and genetic burden. The worldwide prevalence of psoriasis is estimated at approximately 2%, and the prevalence is in a diverse range according to regions (Parisi et al., 2013). Patients with psoriasis have been reported to suffer from elevated rates of various psychopathologies, including depression, anxiety, sexual dysfunction, poor self-esteem, and even suicidal ideation (Kimball et al., 2005; Kimball et al., 2012).

In the past decade, breakthroughs in the understanding of the pathogenesis of psoriasis have developed from its histopathologic accelerated cell proliferation of keratinocytes to the pathogenesis of chronic inflammatory with a dominant IL-23/Th17 axis (Zheng et al., 2007; Ogawa et al., 2018). The crosstalk within neutrophils, dendritic cells, T cells, and the cytokines released from immune cells are the central pathogenesis of psoriasis progress (Boehncke and Schon, 2015).

However, we still lack a holistic knowledge of the role of the myeloid cell-derived immune system in psoriasis. Myeloid-derived suppressor cells (MDSCs) are a heterogeneous population of activated myeloid precursors and relatively immature myeloid cells that are associated with various pathological immune environments (Brandau et al., 2013; Ost et al., 2016). Most studies have divided MDSCs into two major subsets: monocytic myeloid-derived suppressor cells (M-MDSCs) and polymorphonuclear or granulocytic myeloid-derived suppressor cells (PMN-MDSCs or G-MDSCs). PMN-MDSCs are similar to neutrophils, whereas M-MDSCs share many morphological and phenotypic characteristics of monocytes (Gabrilovich and Nagaraj, 2009; Gabrilovich, 2017).

Recent studies have indicated that the MDSC population increases in patients with psoriasis (Cao et al., 2016; Soler et al., 2016). But we still know little about the perturbations of MDSCs in the immune system of psoriasis patients and its immune response in the psoriatic therapeutic progress. To explore how the different subpopulation of MDSCs in psoriasis patients mediate psoriasis development, we detected the expansion of MDSCs and its subpopulation PMN-MDSCs and M-MDSCs in imiquimod (IMQ)-induced murine psoriatic dermatitis. The results showed that the MDSCs and their subpopulations were also different between psoriatic dermatitis mice and the controls.

Recently, series of clinical trials and systematic reviews have reported that Chinese medicine is effective in treating psoriasis (May et al., 2012; Deng et al., 2013; 2014; Zhang et al., 2014). Yinxieling (PSORI-CM) formula is a Chinese herbal medicine compound preparation with 10 ingredients (*Angelica sinensis (Oliv.) Diels, Paeonia lactiflora Pall., Chloranthus spicatus (Thunb.) Makino, Prunus mume (Siebold) Siebold and Zucc., Rehmannia glutinosa (Gaertn.) DC., Ligusticum striatum DC., Lithospermum erythrorhizon Siebold and Zucc., Curcuma zedoaria (Christm.) Roscoe, Smilax glabra Roxb., Glycyrrhcentera glabra L.*) for psoriasis. This medicine was developed by a well-known Chinese Medicine dermatologist National Medical Master Guo-wei Xuan and formulated according to traditional Chinese medicine theory. The clinical practice in these 20 years showed that PSORI-CM formula has been prescribed extensively for psoriasis therapy and has shown a stable curative effect on not only relieving symptoms of psoriasis but also reducing the relapse rate (Cz and Liu, 2006; Han et al., 2011). To expand the application of Yinxieling, an optimized formula PSORI-CM02 was developed, composed of only five ingredients (*Curcuma longa L., Paeonia lactiflora Pall., Smilax glabra Roxb., Prunus mume (Siebold) Siebold and Zucc.* and *Sarcandra glabra (Thunb.) Nakai*) from the original Yingxieling. In the theory of Traditional Chinese medicine, psoriasis is generally associated with three syndromes: *blood stasis*, *blood heat*, and *blood dryness*. In the acute stage of the pathogenesis of psoriasis, *blood heat* is mainly obstructed on the surface of the lesion. In the chronic stage, *qi* and *blood* deficiency lead to *dryness,* which obstructs the normal nourishment in the skin, as well as *blood stasis,* which prohibits blood flow smoothly in the skin (Lu et al., 2012). Therefore, the therapeutic principle of psoriasis is to activate blood circulation and remove blood stasis. In PSORI-CM02 formula, *Curcuma longa L.* and *Paeonia lactiflora Pall.* help to activate blood circulation, while *Smilax glabra Roxb., Prunus mume (Siebold) Siebold and Zucc.* and *Sarcandra glabra (Thunb.) Nakai* play the roles of removing *blood stasis*. These five ingredients in PSORI-CM02 were found to have positive correlations with pharmacodynamic indicators by using computer systematic pharmacological methods and laboratory experiments (Gu et al., 2018). However, previous studies of PSORI-CM02 are mostly focused on the role of the adaptive immune system in psoriasis. One of our previous studies showed that PSORI-CM02 suppressed allograft rejection by reducing the proliferation of T cells (Lu et al., 2018), implying that the mechanism of T-cell suppression needed to be investigated.

The IMQ-induced mouse model for psoriasiform dermatitis is one of the most widely used mouse models in recent research studies on psoriasis, as psoriasis mediated by the IL-23/Th17 axis can be triggered by this TLR7 agonist (van der Fits et al., 2009). Considering there are many effective components characterized by inducing multitarget effects in Chinese Medicine, whether the myeloid cell is also a target of PSORI-CM02 or not is still uncertain. Therefore, based on the observation of MDSCs and their subpopulation in IMQ-induced murine psoriatic dermatitis, we investigated the effect of PSORI-CM02 on MDSCs in the psoriatic mouse model.

Currently, various immunosuppressive and promoting functions of MDSCs on diseases have been reported in the literature. In this study, we performed the functional analysis of murine MDSCs in psoriatic dermatitis. We also discussed the association of MDSCs with T-help cells in psoriasis and the Chinese medicine formula PSORI-CM02 effects on their crosstalk. We aimed at providing suggestions for the contemporary biological characterization of MDSCs and the mechanism of Chinese medicine on psoriasis.

## Materials and Methods

### Reagents and Antibodies

Reagents were purchased as follows: liberase TM (5401119001) was purchased from Roche, DNase I (D4263-1VL) was purchased from Sigma-Aldrich. Cell Staining Buffer (554656), BD GolgiPlug™ (brefeldin A, 550583), RBC Lysing Buffer (10X concentrate), Ms CD11B FITC (561688), Ms Gr1 PE (553128), Ms LY-6G PE (561104), Ms Ly-6C PerCP-Cy™5.5 (560525), Ms CD16/CD32 PURE (553142) and mouse Th1-Th2-Th17 CBA KIT (560485) was purchased from BD (United States), Ms Arg1 (A1exF5) APC (17-3697-80) antibody was purchased from Invitrogen, Arg1 ELISA Kit, TGF-beta1 ELISA Kit, iNOS ELISA Kit, IL-17A ELISA Kit were purchased from Cusabio (China). Mouse CD4^+^ T cell isolation kit II (130-095-248) were purchased from Miltenyi Biotec. Fetal bovine serum (FBS) and High-glucose Dulbecco’s modified Eagle’s medium (DMEM) were purchased from Gibco (United States). Trizol reagent was purchased from Life (United States). RNeasy fibrous tissue mini kit was purchased from Qiagen (Germany), Ms CD45.2 PerCP, Ms CD4 eFluor 660, Ms IL-17A FITC, Ms IL-22 APC, First Strand cDNA Synthesis Kit was purchased from and FastStart Universal SYBR Green Master Mix were purchased from Thermo (United States). RIPA Buffer was purchased from Cell Signaling Technology (United States). Imiquimod cream was obtained from Ming Xin Pharmaceutical Co. LTD. (China), Vaseline® pure petroleum jelly was purchased from Unilever (United Kingdom), methotrexate (MTX) was purchased from the Shanghai Pharmaceutical Group Co. Ltd. (China), PSORI-CM02 was obtained from the Chinese Medicine Hospital of Guangdong Province (China).

BD FACSAria™ III (BD Biosciences, United States), ABI7500 Real-Time PCR System (ABI, United States), NanoDrop 2000C Spectrophotometer (Thermo Scientific, United States), microscope (Olympus, Japan) and VICTOR™ X5 (PerkinElmer, United States) were used for the analysis.

### IMQ-Induced Psoriatic Dermatitis in Mice

BALB/c mice with an equal sex ratio at 6–8 weeks of age with an average weight of 16–18 g were purchased and transferred from Guangdong Medical Laboratory Animal Center (Guangdong, China). All of the mice were settled in a specific pathogen-free (SPF) animal facility with unlimited access to food and water and a 12-h daylight, 12-h night cycle. Mice in the IMQ group received a topical dose of 60 mg imiquimod cream on the shaved back skin (2 × 3 cm) once per day for seven consecutive days, and mice in the control group received Vaseline® as vehicle controls.

A modified Psoriasis Area Severity Index (PASI) score for measuring the inflammation of psoriatic dermatitis was recorded every day before painting of the cream. Erythema, scaling and thickening of back skin were measured based on 0–4 scales (0, none; 1, slight; 2, moderate; 3, marked; and 4, very marked). Bodyweight was measured daily. At day-8 morning, all mice were anaesthetised with a ketamine/acepromazine mixture (ketamine 100 mg/kg, acepromazine 5 mg/kg) and then weighed, PASI was recorded, and the digital photo was taken. Then, the mice were sacrificed. Skin tissues were collected and processed for flow cytometric analysis. The skin sample was also reserved for histological analysis and gene expression analysis.

### Treatment of Mice

The mice were housed in a stable SPF environment for one week after purchase. For the experiments, they were allocated randomly to different groups.

PSORI-CM02 group: 60 mg imiquimod cream painted on the shaved back skin daily, PSORI-CM02 decoction (5 ml/kg) was administered orally twice per day. The treatment of PRORI-CM02 began one week before the IMQ painting and lasted 14 consecutive days. Considering the ethical statement of Institutional Animal Care and Use Committee of Guangdong Provincial Academy of Chinese Medical Sciences, as all of our previous multiple-dose studies indicated that the treatment with PSORI-CM02 decoction dose with 2.94 g/kg to the mice was the most effective (Chen et al., 2017; Wu et al., 2019; Yue et al., 2019; Li et al., 2020), we prepared the PSORI-CM02 decoction dose only with 2.94 g/kg in this study to reduce and refine the use of animals in research.
MTX group: 60 mg imiquimod cream painted on the shaved back skin daily, MTX 0.65 mg/kg was administered orally twice per day.
Model group: 60 mg imiquimod cream painted on the shaved back skin daily, saline was administered orally twice per day.
Control group: Vaseline® painted on the shaved back skin daily, saline was administered orally twice per day.

### Preparation of PSORI-CM02

All the herbs of the PSORI-CM02 formula were pharmacopeia-grade and purchased from the Department of Pharmacy, Guangdong Provincial Hospital of Chinese Medicine. Five herbs are used in the PSORI-CM02 formula: *Curcuma longa L., Paeonia lactiflora Pall., Smilax glabra Roxb., Prunus mume (Siebold) Siebold and Zucc.* and *Sarcandra glabra (Thunb.) Nakai*, with a weight ratio of 2:3:5:2:5. The types of specimens used in this study were deposited in the herbarium in the School of Chinese Materia Medica, Guangzhou University of Chinese Medicine (GUCM).

Water extracts were then concentrated and dried with a rotary evaporator under vacuum. The PSORI-CM02 decoctions were prepared using the following procedures: herbs were marinated in a pot with filtered water for half an hour. Then, the pot was heated and kept boiling for 15 mins. Then, decocting was done for 30 mins with a small fire. The herbs were decocted twice. The two decoctions were mixed, filtered using a 75-μm filter and stored in a refrigerator at 4 °C. A chromatographic fingerprint for quality control and quantitative analysis of the PSORI-CM02 decoction was performed and reported in our previous study (Supplementary Figure S1; Table S1) (Chen et al., 2017; Wu et al., 2019). All procedures followed the procedures of the China Pharmacopoeia (2010 Edition).

### Single-Cell Preparation From Skin

The skin samples from the mice were cut from the shaved back region. Skin samples were chopped into small pieces with scissors and digested with 2 ml of liberase TM (2.5 mg/ml) containing 0.1% DNase I for 90 min at 37 °C. The cell suspensions were filtered with 40 μm of cell strainer and washed twice with 5 ml of PBS 1% FBS.

Drainage from the lymph nodes was obtained from the abdomens of the mice and ground by the frosted surface of the glass slides. The cell suspensions were filtered with 40 μm of cell strainer and washed with 2 ml of PBS 1% FBS.

### Flow Cytometric Analysis

Immune phenotype characterization of the skin sample was performed by flow cytometry with a 12-colour setup, based on the surface staining and intracellular staining method. A 3-h stimulation of 10^6^ cells/ml suspension at 37 °C with PMA (25 ng/ml)/ionomycin (1 mg/ml) in the presence of Brefeldin-A (10 mg/ml) was performed. Before the staining, the cells were resuspended in 10^6^ cells/100 μL per well in a 96-well plate with 2 μg/ml of anti-CD16/CD32 diluted in PBS 1% FBS and incubated at 4 °C for 15 mins to block non-specific binding to Fc receptors. After the preparation, cells were incubated in the dark at 4 °C for 30 mins for surface staining with the following antibodies: Ms CD11B FITC, Ms Gr1 PE, Ms LY-6G PE, Ms Ly-6C PerCP-Cy™5.5. The cells for intracellular staining were also surface stained with the following antibodies: Ms CD45.2 PerCP, Ms CD4 eFluor 660. Intracellular staining was performed for the cells with the following antibodies: Ms IL-17A FITC, Ms IL-22 APC, Ms Arg1 APC. The stained cells were analysed by flow cytometer BD FACSAria III. Data were analysed by using FlowJo software (Version.10.0.8, Tree Star Ashland, OR). The results were represented as the percentage of positive cell populations.

### Skin Biopsy Histological Analysis

The skin biopsies extracted from the mice were fixed in fresh 4% (w/v) formaldehyde solution for 24 h and then embedded in paraffin. After paraffin embedding, the slides were stained with haematoxylin and eosin (H&E).

### Real-Time Quantitative PCR (RT-qPCR)

Skin biopsies were stored under liquid nitrogen. On the day of the RT-qPCR assay, the biopsies were allowed to thaw, and 30 mg of tissue was homogenized with the aid of a homogenizer in ice-cold Trizol reagent. mRNA was obtained from the back-skin biopsy of all mice with the RNeasy fibrous tissue mini-kit. cDNA was synthesized using the Thermo First Strand cDNA Synthesis Kit. For the qPCR reaction, complementary DNA (cDNA) was amplified with FastStart Universal SYBR Green Master Mix. Quantitative research detection of target nucleic acid sequences was then performed on the ViiA seven Real-Time PCR System.

All target genes were normalized with housekeeping gene GAPDH expression in parallel as the internal control. The relative expression of target genes was calculated by the 2^−ΔΔCT^ method.

### Cytometric Bead Array (CBA) Analysis

Skin biopsies were lyzed with Radio-Immunoprecipitation Assay (RIPA) lysis buffer. Th1/Th2/Th17 cytokine (IL-2, IL-4, IL-6, IL-10, IL-17A, TNF and IFN-γ) secretion in the skin and the serum of mice were detected by cytometric bead array, a flow cytometric bead-based technology for multiplexed assay, by using the mouse Th1-Th2-Th17 CBA kit according to the manufacturer’s technical guidelines and protocols (Morgan et al., 2004). Data were acquired through flow cytometry BD FACSAria™ III. FCAP Array software (V4.0, BD Biosciences) was used to calculate the cytokine levels.

### Enzyme-Linked Immunosorbent Assay (ELISA)

Skin biopsies from the shaved back region were stored under liquid nitrogen. On the day of the ELISA assay, the biopsies were thawed, and 20–50 mg of wet tissue was homogenized with the aid of a homogenizer in ice-cold PBS. The homogenates were centrifuged for 20 mins at 10,000 x g to remove debris and insoluble material, and aliquots of the supernatants were assayed by ELISA. Skin tissue Arg1, TGFβ1, iNOS, IL-13, and IL-22 levels were measured using the commercial ELISA kits according to the manufacturer’s instructions. The 96-well plates were detected using VICTOR™ X multilabel reader at a wavelength of 450 nm.

### Cell Isolation

Whole blood was obtained from the inferior vena cava of anaesthetised mice and then collected by lepirudin tubes from the anaesthetised mice. Red Blood Cell (RBC) Lysis Buffer was used to remove the red blood cells from the blood samples. The CD4^+^ T cells were isolated from the sample with CD4^+^ T cell isolation kit II according to the manufacturer’s instruction. To assess the purity, cells were stained with anti-CD4-eFluor 660 antibody and analysed by flow cytometry. The purity of CD4^+^ T cells reached 97% and was acceptable for the coculture.

MDSCs were isolated from the remaining cells by flow cytometric sorting. For flow cytometric sorting, 1 × 10^7^ cells/mL suspension cells were stained with anti-CD11b-FITC, anti-Ly6G-PE and anti-Ly6C -PerCP-Cy™5.5 antibodies for 30 mins on ice in the staining buffer. After the cells were washed twice with PBS, the cells were then sorted with BD FACSAria™ III.

### CD4^+^ T Cells and MDSCs Coculture

M-MDSC (1 × 10^6^) or PMN-MDSC were plated at 1 × 10^6^ cells per well in a 6-well culture plate in RPMI 1640 medium. CD4^+^ T cells were added to MDSC in the Transwell system. Then, CD4^+^ T cells were activated with anti-CD3/anti-CD28. The coculture system was incubated at 37 °C in a humidified incubator and 5% CO_2_ atmosphere for 24 h. Then, the culture supernatant was collected for the enzyme-linked immunosorbent assay.

### Statistical Analysis

All data were described as the mean with standard deviation (SD). Differences between two groups were analysed with unpaired Student’s *t*-test (two-tailed, assuming equal variance). Correlation analysis was performed with the Spearman method. Correlation heatmap was plotted by R (Version 3.6.0). All statistical analyses were performed and presented using GraphPad Prism software (version 6.0 for windows). Statistically significant difference was indicated when *p* < 0.05.

## Results

### MDSCs Were Elevated in IMQ-Induced Psoriatic Dermatitis

**FIGURE 1:**
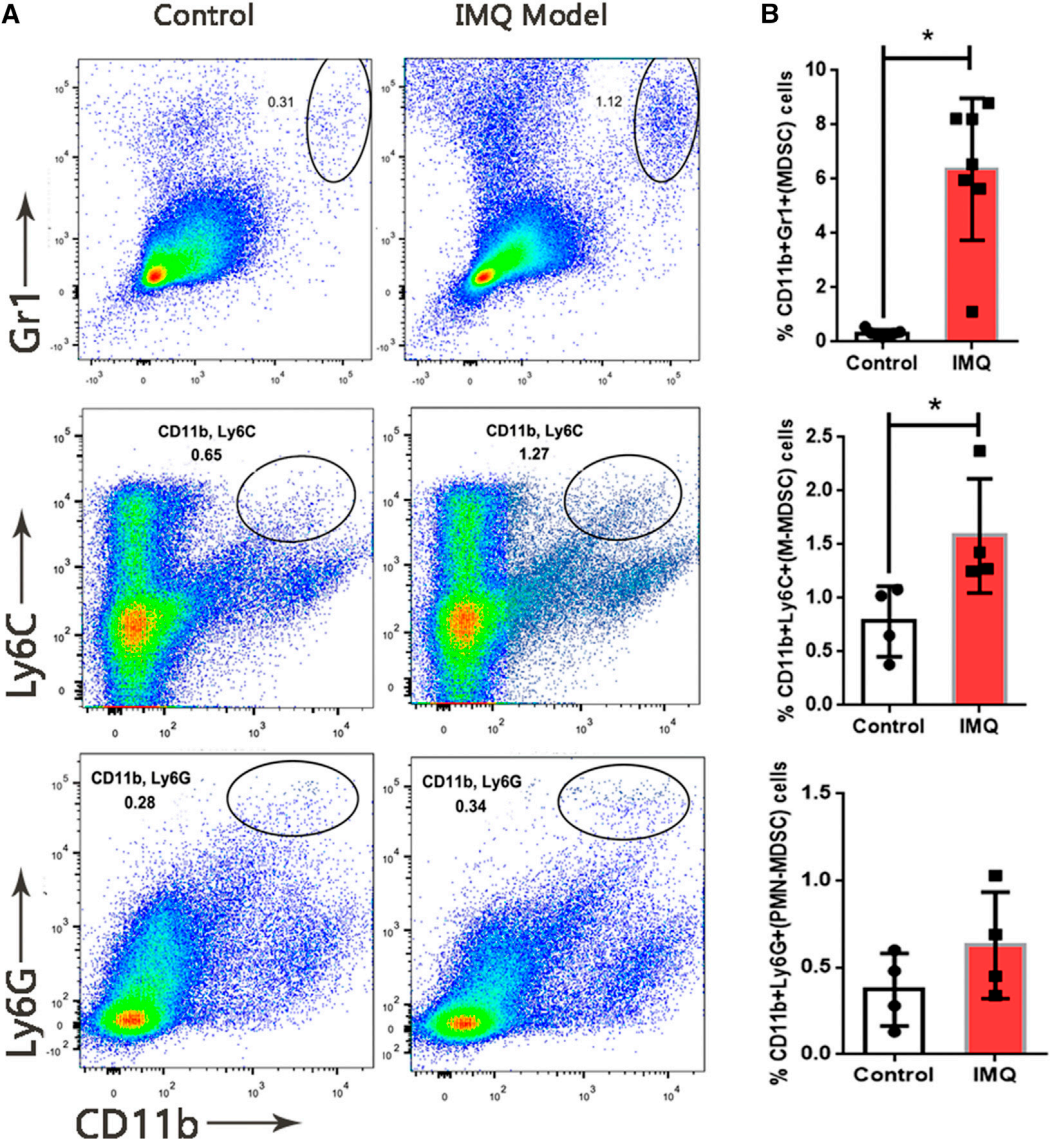
Expansion of MDSCs in IMQ-induced psoriatic dermatitis. **(A)** Flow cytometry analysis of MDSCs (CD11b^+^ Gr1^+^), M-MDSC (CD11b^+^ Ly6C^+^) and PMN-MDSC (CD11b^+^ Ly6G^+^) in skin samples. First row: MDSCs (CD11b^+^ Gr1^+^), second row: M-MDSC (CD11b^+^ Ly6C^+^), third row: PMN-MDSC (CD11b^+^ Ly6C^+^). First column: sample from control group, second column: sample from model (IMQ) group. **(B)** Percentages of MDSCs, M-MDSC and PMN-MDSC in live cells of skin tissue. Seven mice per group in MDSC analysis, four mice per group in M-MDSC and PMN-MDSC analysis; *p < 0.05, **p < 0.01.

**FIGURE 2:**
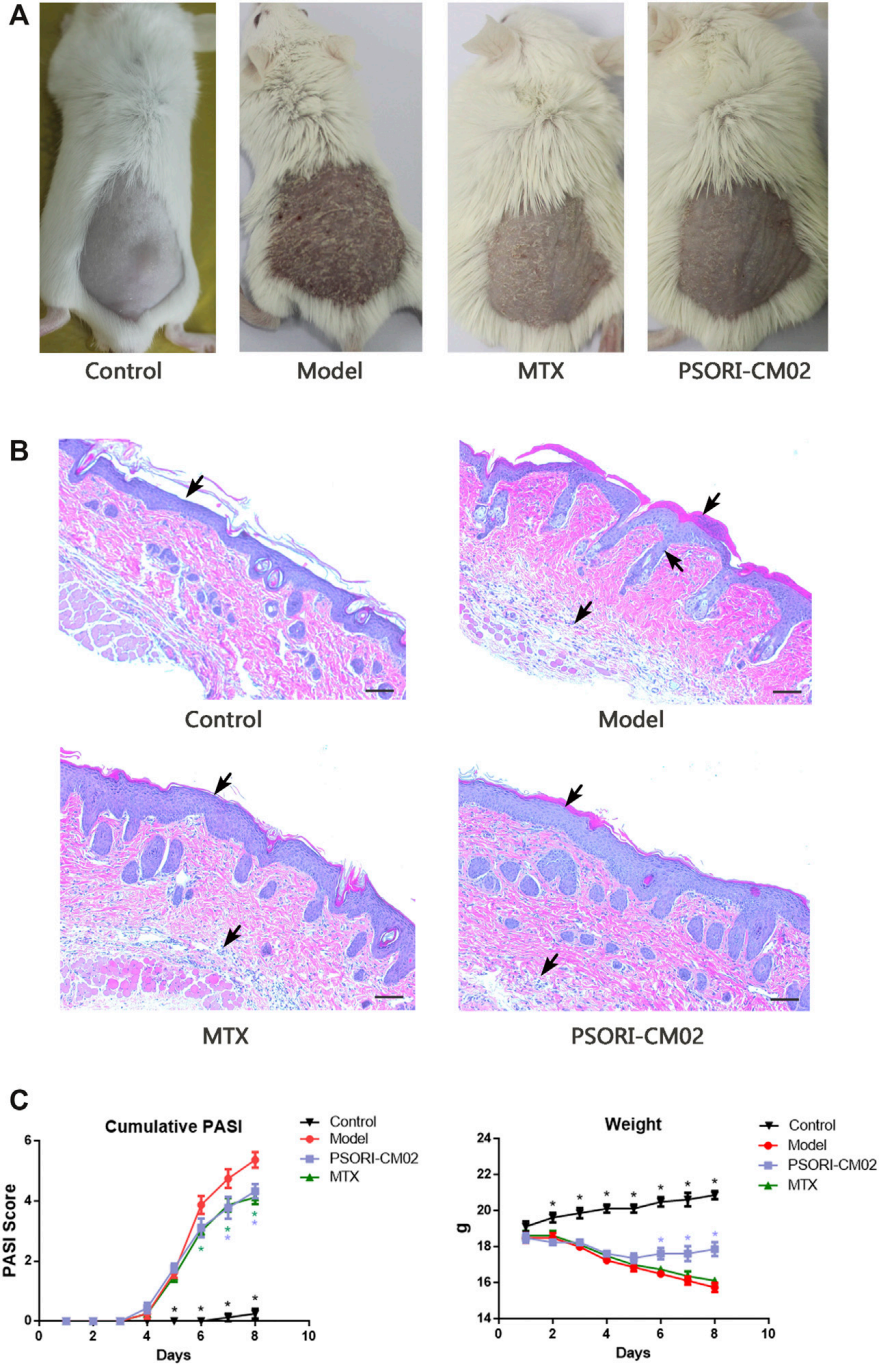
Different treatment caused different effects on IMQ-induced psoriatic dermatitis. **(A)** Skin inflammatory symptoms in different groups. **(B)** H&E stained sections of back skin of mice in different groups. Psoriasis-specific hyperkeratosis, parakeratosis, neovascularization and infiltration of immune cells in dermis and epidermis were labeled by arrows. **(C)** Accumulative PASI score based on the erythema, induration, desquamation and percentage of affected area during the dermatitis progress. *n* = 8 mice per group **(D)** Weight loss during the treatments challenged. *n* = 8 mice per group.

To explore the role of MDSCs in psoriasis, a mouse model with skin inflammation was induced by IMQ to mimic psoriasis (Figure 1A). H&E stained sections of the back skin of mice treated with 5% IMQ cream showed the epidermal thickening due to hyperkeratosis, parakeratosis, neovascularization and infiltration of immune cells in dermis and epidermis (Figure 1B). To determine the dysfunction of MDSCs and its subpopulations in psoriatic dermatitis, we measured the MDSCs marker levels of CD11b, Gr1, Ly-6G and Ly-6C in mouse skin samples by using flow cytometry (Figures 2A,B). The percentages (%) of MDSCs (CD11b^+^ Gr1^+^) and M-MDSCs (CD11b^+^ Ly-6C^+^) among total cells significantly increased in the skin samples of IMQ mice. These results suggested that the MDSCs, especially M-MDSCs, were involved in the pathological mechanism of IMQ-induced psoriatic dermatitis.

### PSORI-CM02 Alleviated IMQ-Induced Psoriatic Dermatitis

Mice in different groups showed different effects on the skin inflammatory symptoms such as the erythema, thickness and scales. Comparing to the model group, dermatitis in the MTX group and the PSORI-CM02 group was alleviated significantly (Figure 1A). H&E stained sections of the back skin of mice in different groups presented a consistency with skin inflammatory symptoms. Hyperkeratosis, parakeratosis, neovascularization and infiltration of immune cells into the dermis and epidermis in the MTX group and PSORI-CM02 group were lighter than those parameters in the model group (Figure 1B).

The accumulative PASI score of the model group at day 7 was 5.38 ± 1.18, whereas the score was 4.13 ± 0.64 in the MTX group and 4.33 ± 0.71 in the PSORI-CM02 group (Figure 1C). Body weights were recorded daily, showing the difference between the PSORI-CM02 group and other groups of those challenged with IMQ. PSORI-CM02 treatment alleviated IMQ-caused weight loss (Figure 1D).

### PSORI-CM02 Reduced M-MDSCs in Psoriatic Dermatitis Tissue and Lymph Nodes

**FIGURE 3:**
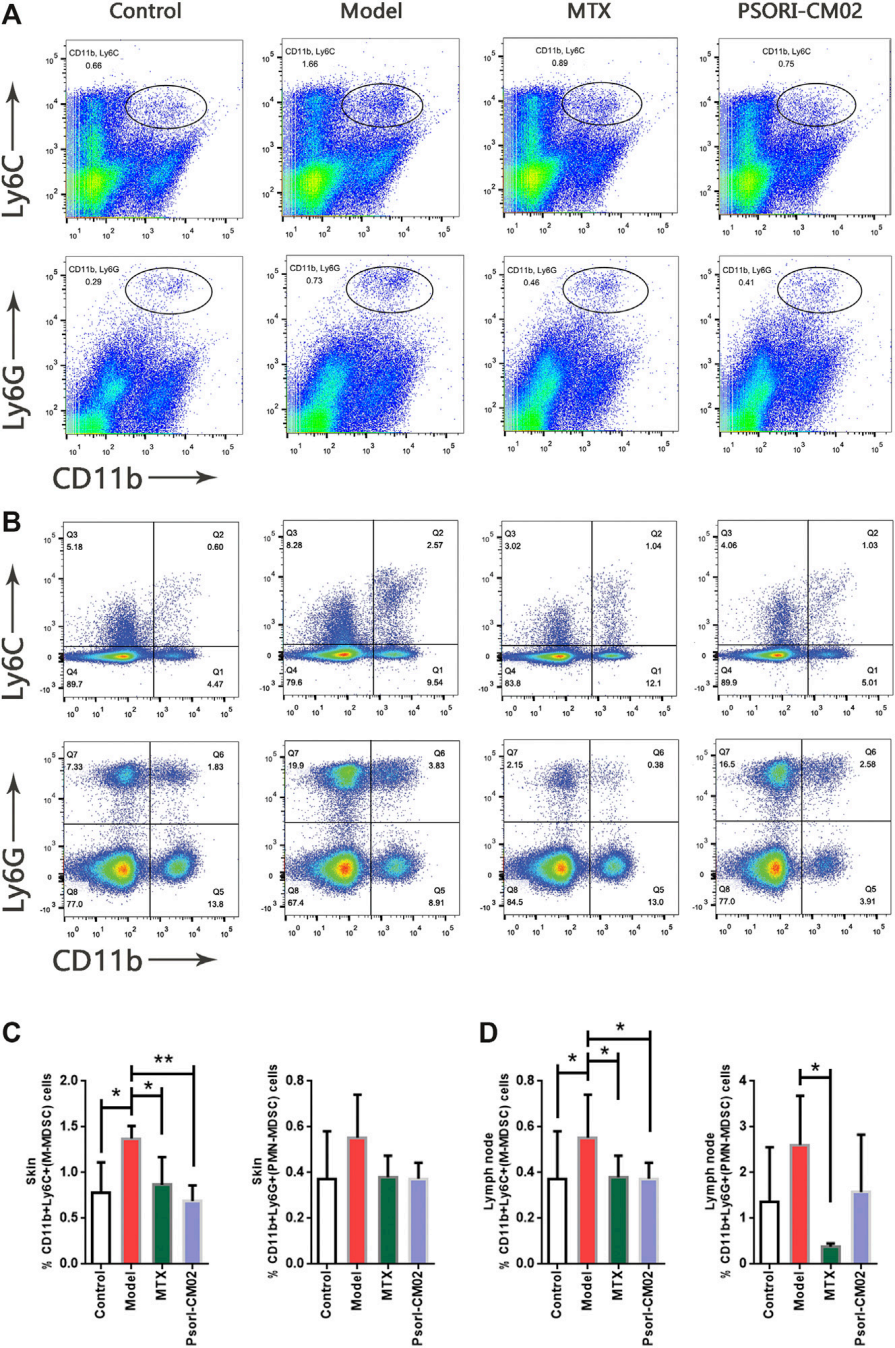
Treatment effects on M-MDSC and PMN-MDSC. **(A)** Flow cytometric analysis on M-MDSC and PMN-MDSC levels in skin samples of different groups. First row: M-MDSC (CD11b^+^ Ly6C^+^), second row: PMN-MDSC (CD11b^+^ Ly6C^+^). **(B)** Flow cytometric analysis on M-MDSC and PMN-MDSC levels in lymph nodes of different groups. First row: M-MDSC (CD11b^+^ Ly6C^+^), second row: PMN-MDSC (CD11b^+^ Ly6C^+^). **(C)** Percentages of M-MDSC and PMN-MDSC in live cells of skin samples of different groups. *n* = 4 mice per group; *p < 0.05, **p < 0.01. **(D)** Percentages of M-MDSC and PMN-MDSC in live cells of lymph nodes of different groups. *n* = 4 mice per group; *p < 0.05.

To determine whether PSORI-CM02 treatment was affected via MDSCs, we evaluated the M-MDSC and PMN-MDSC levels in skin and lymph node samples of different groups by flow cytometric analysis (Figures 3A,B). The results showed that the percentages of M-MDSCs differed among the groups. In the PSORI-CM02 group and the MTX group, the percentage of M-MDSCs was lower than in the model group, not only in skins but also in lymph nodes (Figure 3C). No differences were observed in the PMN-MDSCs in skins from different groups. However, in lymph nodes, MTX can reduce the cell ratio of PMN-MDSCs (Figure 3D). These results were consistent with the conclusion in Figure 2 that M-MDSCs but not PMN-MDSCs played a role in psoriatic dermatitis.

### PSORI-CM02 Reduced the Inflammatory Infiltrate in Psoriatic Dermatitis Tissue

**FIGURE 4:**
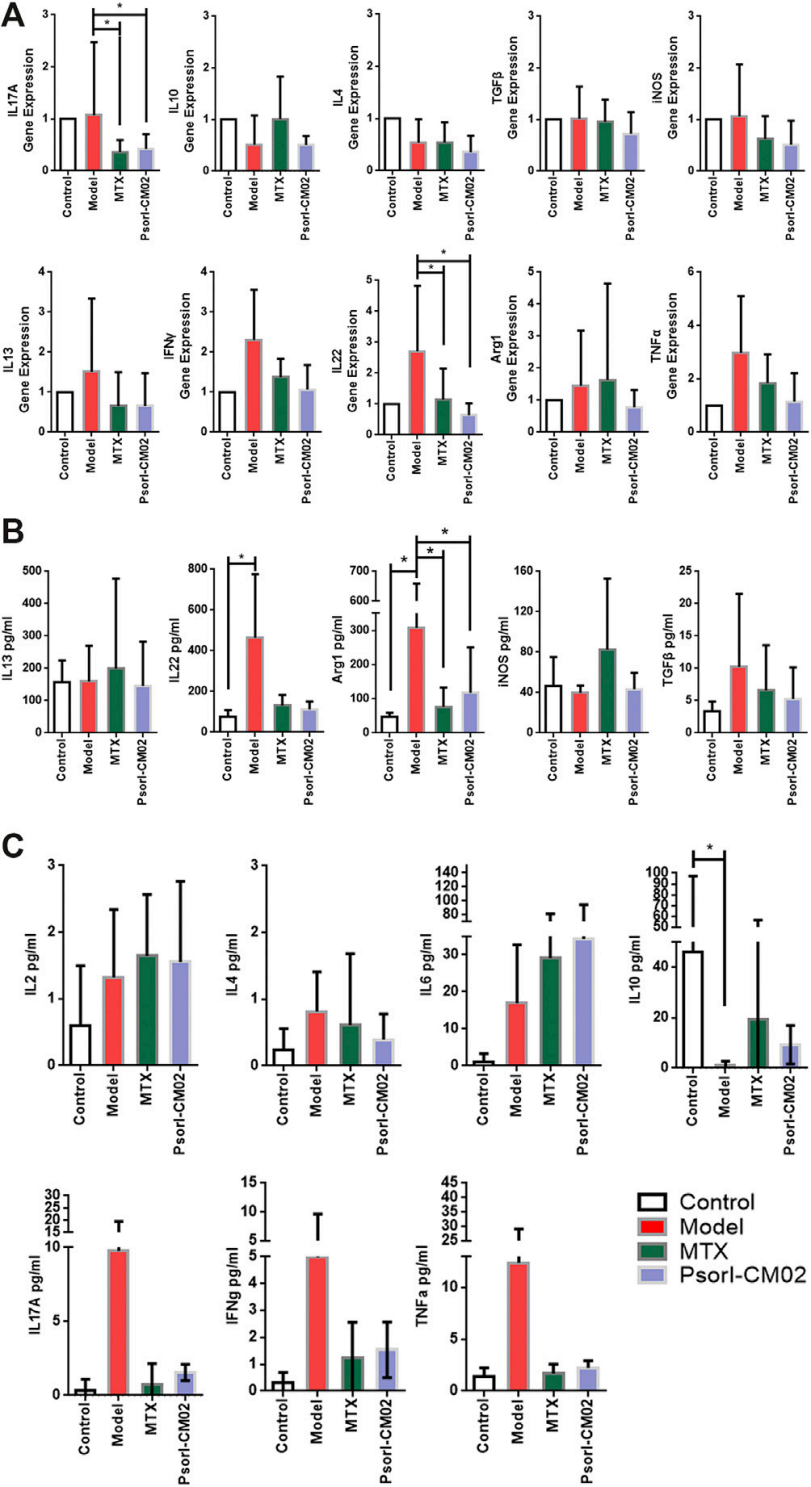
MDSCs and psoriasis related functional characterization by different assays. **(A)** Genes expression of M-MDSC markers and psoriatic inflammatory cytokine in different groups, detected by RT-qPCR. *n* = 5 mice per group; Compared to model group, *p < 0.05. **(B)** M-MDSC markers and psoriasis related inflammatory cytokine secretion in back skin of mice from different groups, detected by ELISA. *n* = 5 mice per group; Compared to model group, *p < 0.05. **(C)** Th1/Th2/Th17 related inflammatory cytokine secretion in back skin of mice from different groups, detected by CBA. *n* = 5 mice per group; Compared to model group, *p < 0.05.

To analyse the mechanism of PSORI-CM02 treatment effected on the psoriatic immune environment, we evaluated the M-MDSC markers (Arg1, TGFβ1, iNOS, IL-10, and IL-13) and psoriatic inflammatory cytokines (Th1/Th2/Th17 cytokines) in skin samples of different groups by CBA, ELISA and RT-qPCR. The results acquired from different assays are concisely illustrated in Figure 4.

As expected, at the transcript level, the psoriatic inflammatory cytokines IL-17 and IL-22 increased in the model group and decreased notably after MTX and PSORI-CM02 (Figure 4A). At the protein level, Arg1, IL-10 and IL-22 were significantly different between model group and control. Only Arg1 changed significantly after treatments (Figures 4B,C).

**FIGURE 5:**
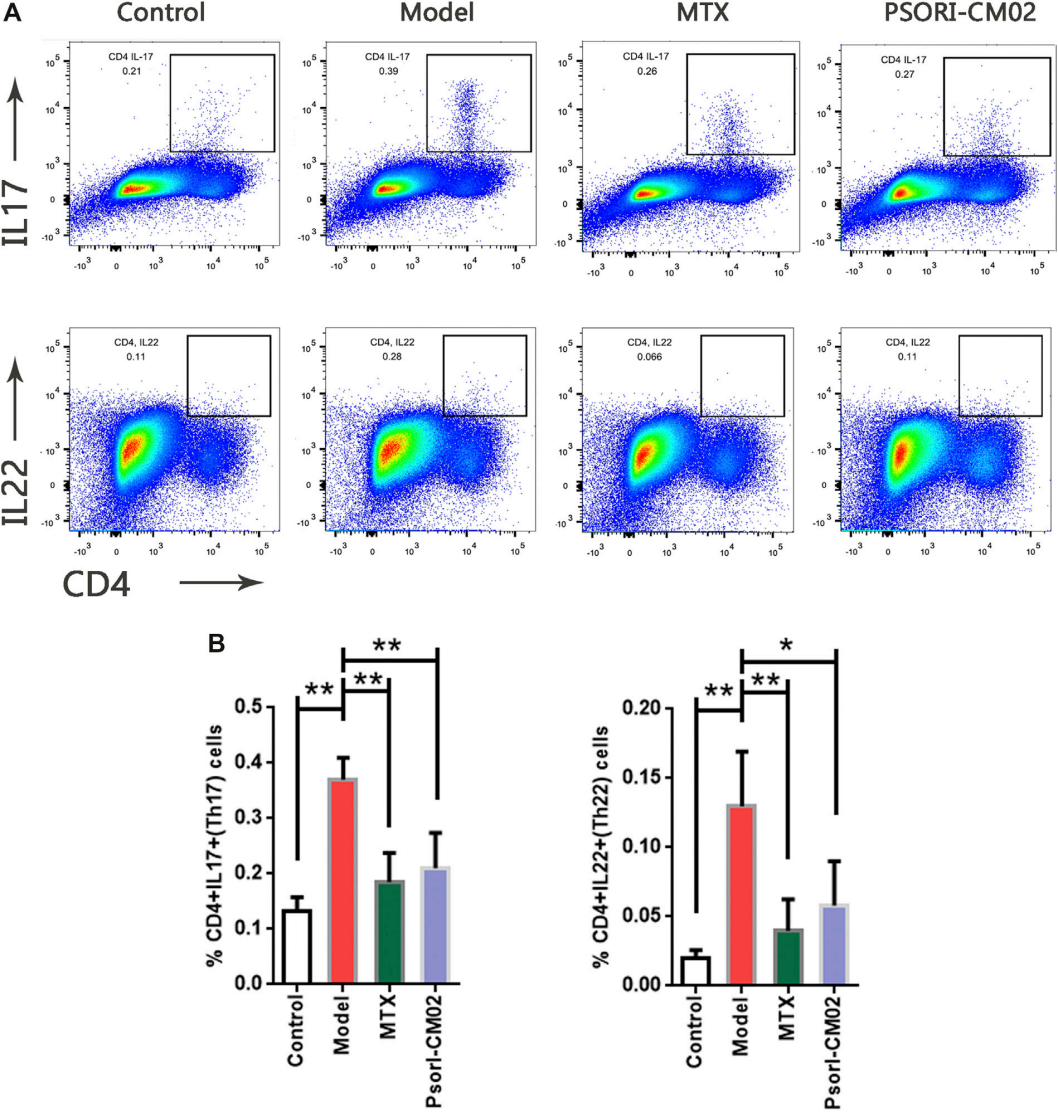
Expansion of IL-17^+^ CD4^+^ T cells and IL-22^+^ CD4^+^ T cells in different groups. **(A)** Flow cytometric analysis on IL-17^+^ CD4^+^ T cells and IL-22^+^ CD4^+^ T cells in live cells of back skin of mice from different groups. First row: IL-17^+^ CD4^+^ T cells, second row: IL-22^+^ CD4^+^ T cells. **(B)** Percentages of IL-17^+^ CD4^+^ T cells and IL-22^+^ CD4^+^ T cells in live cells of back skin of mice from different groups. *n* = 4 mice per group; *p < 0.05, **p < 0.01.

Given that IL-17 and IL-22 positively related to psoriatic dermatitis at the mRNA level but not at the protein level detected by CBA, we examined the IL-17^+^CD4^+^ T cells and IL-22^+^CD4^+^ T cells in the skin by flow cytometric analysis (Figure 5A). The results showed that the percentages of IL-17^+^CD4^+^ T cells and IL-22^+^CD4^+^ T cells differed among the groups. Compared to the control, they were both higher in the model group. MTX and PSORI-CM02 reduced the percentages of IL-17^+^CD4^+^ T cells and IL-22^+^CD4^+^ T cells (Figure 5B). These results indicated that PSORI-CM02 reduced IL-17 and IL-22 mainly to control the inflammatory infiltrate in psoriatic dermatitis.

### Relationship of MDSCs and Psoriatic Inflammatory Cytokines

**FIGURE 6:**
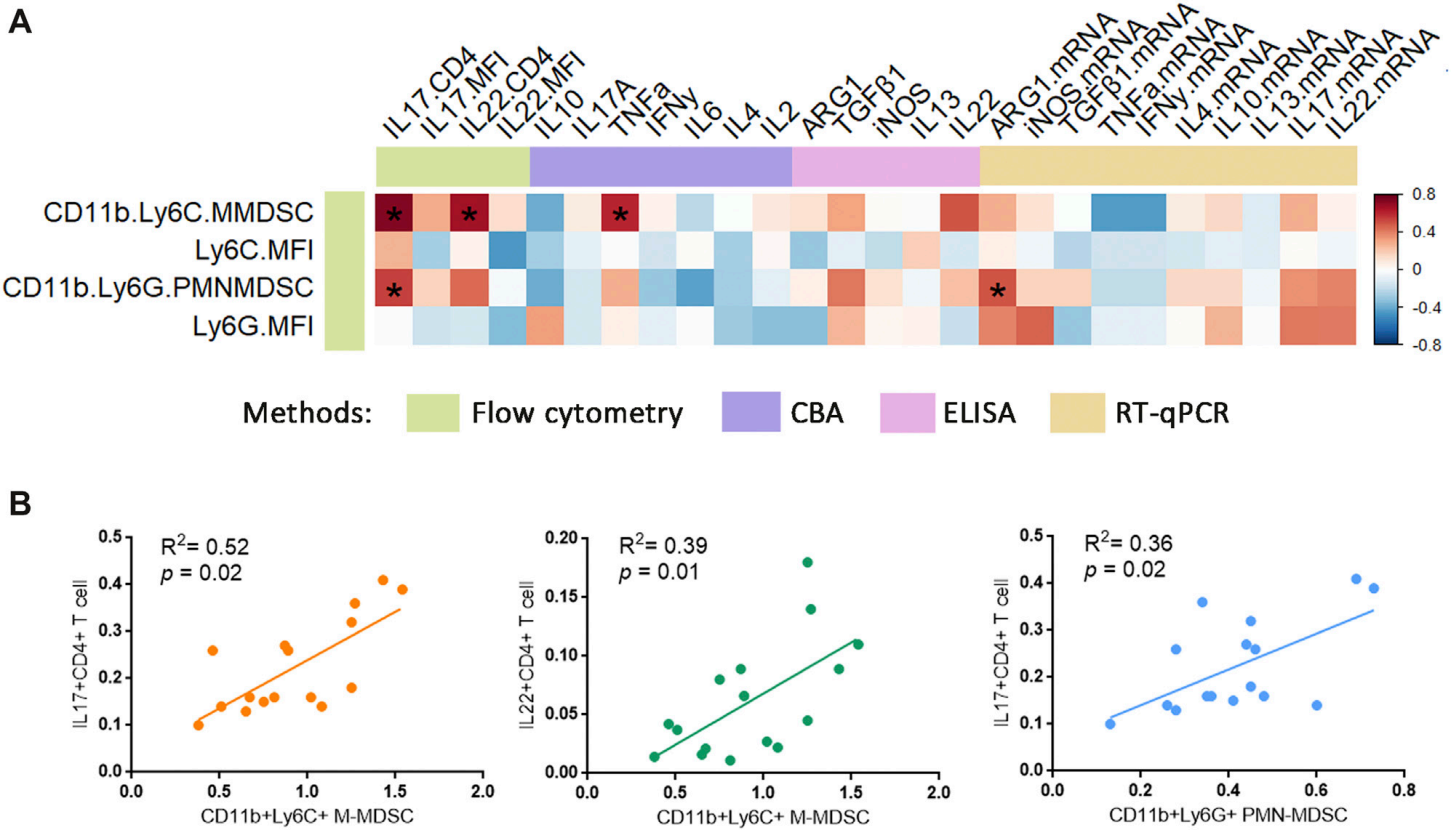
Relationship of MDSCs with psoriatic inflammatory cytokine. **(A)** Correlation heatmap between MDSCs and psoriatic inflammatory cytokine in all samples were analysed using Spearman method. For correlation analysis, ΔCt was used as related gene expression value. Colour bar labelled the different methods/technologies for acquiring the data. Colour scale (right) indicated the r value. *p < 0.05. **(B)** Correlations between % CD11b^+^ Ly6C^+^ MDSCs and % IL-17^+^ CD4^+^ T cells, % CD11b^+^ Ly6C^+^ MDSCs and % IL-22^+^ CD4^+^ T cells, % CD11b^+^ Ly6G^+^ MDSCs and % IL-17^+^ CD4^+^ T cells in all samples were analysed using Spearman’s coefficient.

The above results showed that PSORI-CM02 reduced both M-MDSCs and inflammatory cytokines IL-17 and IL-22. However, the evidence for the relationship of MDSCs and psoriatic inflammatory cytokines *in vivo* was still lacking. To clarify that point, we performed a correlation analysis on MDSC levels and inflammatory cytokines. The heatmap depicts the full-scale statistical analysis using all samples (Figure 6A), revealing a very strong correlation between M-MDSCs and IL-17^+^CD4^+^ T cells, the same as the IL-22^+^CD4^+^ T cells. PMN-MDSC was positively correlated to IL-17^+^CD4^+^ T cells as well (Figure 6B).

### Psoriatic MDSCs Promoted Th17 Cell Differentiation via Arg1

**FIGURE 7:**
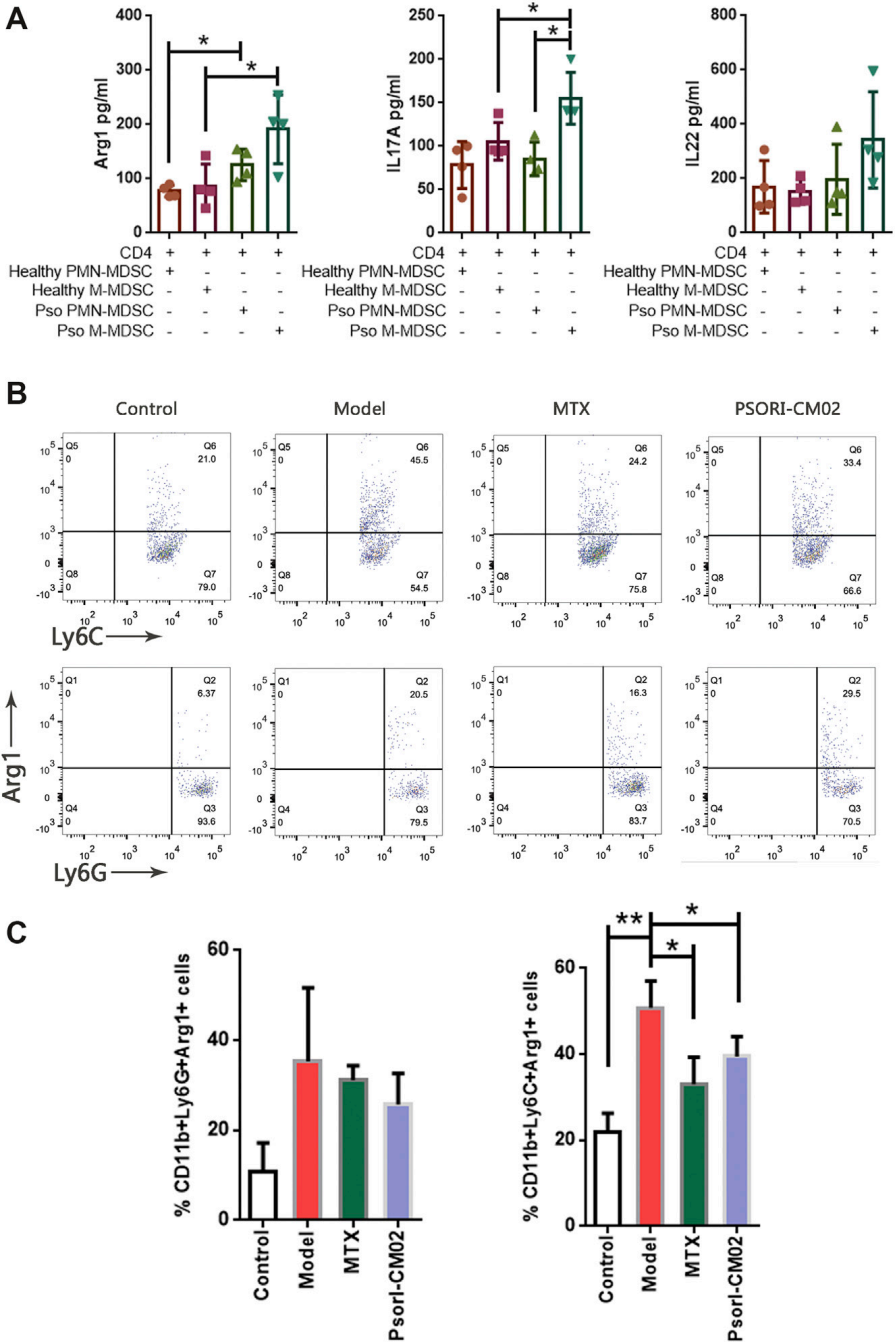
MDSCs induced Th17 differentiation. **(A)** The expression of Arg1, IL-17A and IL-22 in the co-culture supernatant. *p < 0.05. **(B)** Flow cytometric analysis on Arg1+ M-MDSC and Arg1+ PMN-MDSC in skin samples of different groups. First row: Arg1+ M-MDSC cells, second row: Arg1+ PMN-MDSC T cells. **(C)** Percentages of Arg1+ M-MDSC and Arg1+ PMN-MDSC in different groups. *n* = 3 in control group and *n* = 4 in other groups; *p < 0.05, **p < 0.01.

We screened the gene expression and protein abundance of MDSCs and psoriasis-related molecules (Figure 4). The correlation of MDSCs and Th17 was confirmed (Figure 6). Only Arg1, IL-17, and IL-22 were positive in the above assays. We then assessed the effect of MDSCs on anti-CD3/CD28-activated CD4^+^ T cells in the coculture system. After the CD4 T cells and MDSCs coculture, the secretions of Arg1 by M-MDSC or PMN-MDSC obtained from IMQ mice were higher than the secretions from normal mice, suggesting that the effect of MDSC subpopulations is mediated by their secretion of Arg-1. In addition, M-MDSC obtained from IMQ mice can significantly promote the secretion of IL-17A by CD4^+^ T cells (Figure 7A), implying that M-MDSC from IMQ mice was much more potent in Th17 cell polarisation via Arg1. When we compared the secretions of Arg1 by M-MDSC and PMN-MDSC from different treatments, we found that both MTX and PSORI-CM02 could significantly reduce the production of Arg1 from M-MDSC but not from PMN-MDSC (Figures 7B,C).

## Discussion

Psoriasis is a chronic inflammatory disease characterized by erythema, with thickening and scaling of the skin. Its histopathologic change in the epidemic is abnormal proliferation and differentiation of keratinocyte (Boehncke, 2015; Boehncke and Schon, 2015). However, the cause of this histopathologic change remains unknown. Accumulating evidence indicates that psoriasis is a T-cell-mediated disease (Gottlieb et al., 1995). The IL-23/Th17 pathway plays a pivotal role in the progress of psoriasis (Hawkes et al., 2017). The Th17 cell is the key factor in the inflammatory infiltrate, whereas the development of Th17 is maintained by IL-23, which is secreted mainly by monocytes and dendritic cells. With the dendritic cell activation, lymphocytes, neutrophils and monocytes migrate into the skin. Th17 cells produce inflammatory cytokines, including IL-17 and IL-22. IL-17 and IL-22 lead to hyperkeratosis and parakeratosis. TNFα production is induced indirectly by IL-17 and IL-22, which accelerates the inflammatory infiltrate. (Zheng et al., 2007; Ogawa et al., 2018). In clinical practice, biologics targeting these cytokines showed credible efficacy on psoriasis, which proved the importance of the role of the IL-23/Th17 axis in the aetiology of psoriasis.

Recently, the important biological role of MDSCs has been investigated. MDSCs are a cluster of heterogeneous cells that present immunosuppressive to T cell and NK cell function (Gabrilovich and Nagaraj, 2009; Dar et al., 2020). The absence of MDSCs caused T cell proliferation, whereas the accumulation of MDSCs was associated with *tuberculosis* (TB) progress and severity (Knaul et al., 2014; Tsiganov et al., 2014). Myeloid-derived cells have been indicated as an essential factor in the immune system, particularly in cancer immunotherapy. Two subtypes of MDSCs have clear roles that have been established in murine: CD11b^+^Ly6G-Ly6C^+^ monocytic MDSCs (M-MDSCs) and CD11b^+^Ly6G^+^Ly6C- polymorphonuclear or granulocytic myeloid-derived suppressor cells (PMN-MDSCs or G-MDSCs) (Wu and Chiang, 2019). Generally, PMN-MDSCs make up the majority of the population of MDSCs. Both M-MDSCs and PMN-MDSCs have strong immunosuppressive activity, which has been indicated to contribute to immunosuppression in the tumour microenvironment and allergic inflammation (Gabrilovich and Nagaraj, 2009). Meanwhile, both PMN-MDSCs and M-MDSCs can reverse from being immunosuppressive to immunostimulatory. The proportion of subpopulations of MDSCs is highly variable (Ben-Meir et al., 2018). In response to different conditions or environments, M-MDSCs can directionally differentiate into dendritic cells or macrophages, while PMN-MDSCs consist of myeloid progenitors that differentiate into granulocytes (Kumar et al., 2016).

In recent years, CD14^+^HLA-DR-/low M-MDSCs have been reported to suppress T-cell activation (Lauret Marie Joseph et al., 2020). In psoriatic patient PBMC, M-MDSCs was increased, compared to the healthy control (Soler et al., 2016; Sun et al., 2020). *In vivo* and *in vitro* assays demonstrated that Treg cells induced by M-MDSCs in the PBMC of a psoriasis patient presented suppressive functionality reduction. These results suggest that M-MDSCs increased in psoriasis with the impaired suppressive function of effector T-cell expansion. Our present study also proved that MDSCs, especially M-MDSCs, were elevated in IMQ-induced psoriatic dermatitis and lymph nodes.

As Th17 has been found to increase in psoriatic dermatitis, it would be interesting to understand how MDSCs interact with T cells in the immune environment of psoriasis. The functions of MDSCs in suppressing T cells, including producing inducible arginase-1 (Arg1) and inducible nitric oxide synthase (iNOS), which lead to the nitration of T cell receptors and chemokines that are essential for T cell migration, induce T cell apoptosis (Gabrilovich et al., 2012). IL-10 and TGF-β1 are produced, inhibiting immune effector cell proliferation and functions (Umansky et al., 2017). We therefore checked all these molecules in the present study. However, only Arg1 was significantly different between IMQ-induced psoriatic dermatitis and control, and Arg1 was reduced after treatment. The dynamic changes in Arg1 for different groups strongly suggested that M-MDSC was involved in the pathogenesis of psoriasis. However, the functional characterisation presented that most of the marker molecules of MDSCs showed negative results. The relationship between MDSCs and Th17 cells was confirmed by the correlation analysis. Both M-MDSCs and PMN-MDSCs were positively correlated to IL-17^+^CD4^+^ T cells, while M-MDSCs were also associated with IL-22^+^CD4^+^ T cells.

PSORI-CM02 is the Chinese medicine formula formulated based on the Chinese medicine theory (Blood Stasis). As a compound formula, PSORI-CM02 exerts its effects via the combination of the five herbal components. According to our prior published results, PSORI-CM02 can alleviate psoriatic dermatitis induced by IMQ in mice, by increasing the regulatory T cell level (Chen et al., 2017), inducing autophagy to promote the apoptosis of keratinocytes (Yue et al., 2019), and regulating the infiltration and polarisation of macrophages (Li et al., 2020). We found that PSORI-CM02 alleviated IMQ-induced psoriatic dermatitis and suppressed proliferation of M-MDSCs and Th17 cells. Interestingly, M-MDSCs-induced Arg1 is confirmed to cause the suppression of T cell by depletion of the semi-essential amino acid l-arginine (Bogdan, 2011). MDSCs are reported to induce the suppressive activity of Th17 cells through the upregulation of Arg1 (Wu et al., 2016). In our study, the correlation analysis indicated that MDSCs and Th17 cells had a strong relationship with each other, suggesting the existence of crosstalk between MDSCs and Th17 cells in dermatitis. This crosstalk was involved in the therapeutic progress of PSORI-CM02. However, the positive correlation does not mean that the change in MDSCs or Th17 is the cause of the change in the levels of the other variable. To establish the relationship between MDSC and Th17 and the regulatory effects of PRORI-CM02 in the psoriatic dermatitis model, we assessed the *in vitro* coculture assay and found that M-MDSC from IMQ mice was much more potent in Th17 cell polarisation via Arg1. *In vivo* experiment indicated that PSORI-CM02 could significantly reduce the production of Arg1 from M-MDSC, which implied that PSORI-CM02 suppressed proliferation of Th17 cells by targeted M-MDSC-induced Arg1.

## Conclusion

Taken together, our data provided evidence that the percentage of CD11b^+^ Ly6C^+^ M-MDSCs was elevated in IMQ-induced psoriatic dermatitis. Chinese medicine formula PSORI-CM02 alleviated IMQ-induced psoriatic dermatitis and suppressed proliferation of M-MDSCs and Th17 cells. Moreover, our study also determined that M-MDSCs were positively associated with Th17 cell. Psoriatic MDSCs promoted Th17 cell differentiation via Arg1, suggesting that PSORI-CM02 exerted its effects on suppressing Th17 cells by targeted M-MDSC-induced Arg1.

## Data Availability Statement

The raw data supporting the conclusions of this article will be made available by the authors, without undue reservation.

## Ethics Statement

The animal study was reviewed and approved by Institutional Animal Care and Use Committee of Guangdong Provincial Academy of Chinese Medical Sciences.

## Author Contributions

CL and LH designed the study and revised the manuscript. JD, RL, WY, HC, YX, ST and NT performed the experiments, JD conducted the data analysis and drafted the manuscript. All authors have read and approved the final manuscript.

## Funding

This study was supported by the National Natural Science Foundation of China (No. 81603619), Science and Technology Planning Project of Guangdong Province (No. 2017B030314166 and 2020B1111100006); Natural Science Foundation of Guangdong Province (No. 2020A1515010607) and Special Funding for TCM Science and Technology Research of Guangdong Provincial Hospital of Chinese Medicine (No. YN2019QL12 and YN2018RBA02).

## Conflict of Interest

The authors declare that the research was conducted in the absence of any commercial or financial relationships that could be construed as a potential conflict of interest.

## References

[B1] Ben-Meir K. Twaik N. Baniyash M. (2018). Plasticity and biological diversity of myeloid derived suppressor cells. Curr. Opin. Immunol. 51, 154–161. 10.1016/j.coi.2018.03.015 29614426

[B2] Boehncke W. H. Schön M. P. (2015). Psoriasis. Lancet 386 (9997), 983–994. 10.1016/S0140-6736(14)61909-7 26025581

[B3] Boehncke W. H. (2015). Etiology and pathogenesis of psoriasis. Rheum. Dis. Clin. N. Am. 41 (4), 665–675. 10.1016/j.rdc.2015.07.013 26476225

[B4] Bogdan C. (2011). Regulation of lymphocytes by nitric oxide. Methods Mol. Biol. 677, 375–393. 10.1007/978-1-60761-869-0_24 20941622

[B5] Brandau S. Moses K. Lang S. (2013). The kinship of neutrophils and granulocytic myeloid-derived suppressor cells in cancer: cousins, siblings or twins? Semin. Canc. Biol. 23 (3), 171–182. 10.1016/j.semcancer.2013.02.007 23459190

[B6] Cao L. Y. Chung J. S. Teshima T. Feigenbaum L. Cruz P. D. Jacobe H. T. (2016). Myeloid-derived suppressor cells in psoriasis are an expanded population exhibiting diverse T-cell-suppressor mechanisms. J. Invest. Dermatol. 136 (9), 1801–1810. 10.1016/j.jid.2016.02.816 27236103 PMC4992618

[B7] Chen H. Liu H. Lu C. Wang M. Li X. Zhao H. (2017). PSORI-CM02 formula increases CD4+ Foxp3+ regulatory T cell frequency and ameliorates imiquimod-induced psoriasis in mice. Front. Immunol. 8, 1767 10.3389/fimmu.2017.01767 29358932 PMC5766646

[B8] Cz L. X. W. Liu F. N. (2006). Effect of Yinxieling on PCNA expressionand apoptosis of keratinocyte *Trad* . Chin Drug Res Clin Pharmacol. 17, 329–331.

[B9] Dar A. A. Patil R. S. Pradhan T. N. Chaukar D. A. D’Cruz A. K. Chiplunkar S. V. (2020). Myeloid-derived suppressor cells impede T cell functionality and promote Th17 differentiation in oral squamous cell carcinoma. Cancer Immunol. Immunother 69 (6), 1071-1086. 10.1007/s00262-020-02523-w 32103293 PMC11027600

[B10] Deng S. May B. H. Zhang A. L. Lu C. Xue C. C. (2013). Plant extracts for the topical management of psoriasis: a systematic review and meta-analysis. Br. J. Dermatol. 169 (4), 769–782. 10.1111/bjd.12557 23909714

[B11] Deng S. May B. H. Zhang A. L. Lu C. Xue C. C. (2014). Topical herbal formulae in the management of psoriasis: systematic review with meta-analysis of clinical studies and investigation of the pharmacological actions of the main herbs. Phytother Res. 28 (4), 480–497. 10.1002/ptr.5028 23817996

[B12] Gabrilovich D. I. Nagaraj S. (2009). Myeloid-derived suppressor cells as regulators of the immune system. Nat. Rev. Immunol. 9 (3), 162–174. 10.1038/nri2506 19197294 PMC2828349

[B13] Gabrilovich D. I. Ostrand-Rosenberg S. Bronte V. (2012). Coordinated regulation of myeloid cells by tumours. Nat. Rev. Immunol. 12 (4), 253–268. 10.1038/nri3175 22437938 PMC3587148

[B14] Gabrilovich D. I. (2017). Myeloid-derived suppressor cells. Cancer Immunol. Res. 5 (1), 3–8. 10.1158/2326-6066.CIR-16-0297 28052991 PMC5426480

[B15] Gottlieb S. L. Gilleaudeau P. Johnson R. Estes L. Woodworth T. G. Gottlieb A. B. (1995). Response of psoriasis to a lymphocyte-selective toxin (DAB389IL-2) suggests a primary immune, but not keratinocyte, pathogenic basis. Nat. Med. 1 (5), 442–447. 10.1038/nm0595-442 7585092

[B16] Gu J. Li L. Wang D. Zhu W. Han L. Zhao R. (2018). Deciphering metabonomics biomarkers-targets interactions for psoriasis vulgaris by network pharmacology. Ann. Med. 50 (4), 323–332. 10.1080/07853890.2018.1453169 29537306

[B17] Han L. Peng Y. Zhao R. Z. Feng B. Lu C. J., (2011). Effect of yinxieling on proliferation of HaCaT. J. Guanghou Univ. TCM. 28, 159–162.

[B18] Hawkes J. E. Chan T. C. Krueger J. G. (2017). Psoriasis pathogenesis and the development of novel targeted immune therapies. J. Allergy Clin. Immunol. 140 (3), 645–653. 10.1016/j.jaci.2017.07.004 28887948 PMC5600287

[B19] Kimball A. B. Jacobson C. Weiss S. Vreeland M. G. Wu Y. (2005). The psychosocial burden of psoriasis. Am. J. Clin. Dermatol. 6 (6), 383–392. 10.2165/00128071-200506060-00005 16343026

[B20] Kimball A. B. Wu E. Q. Guérin A. Yu A. P. Tsaneva M. Gupta S. R. (2012). Risks of developing psychiatric disorders in pediatric patients with psoriasis. J. Am. Acad. Dermatol. 67 (4), 651–652. 10.1016/j.jaad.2011.11.948 22243764

[B21] Knaul J. K. Jörg S. Oberbeck-Mueller D. Heinemann E. Scheuermann L. Brinkmann V. (2014). Lung-residing myeloid-derived suppressors display dual functionality in murine pulmonary tuberculosis. Am. J. Respir. Crit. Care Med. 190 (9), 1053–1066. 10.1164/rccm.201405-0828OC 25275852

[B22] Kumar V. Patel S. Tcyganov E. Gabrilovich D. I. (2016). The nature of myeloid-derived suppressor cells in the tumor microenvironment. Trends Immunol. 37 (3), 208–220. 10.1016/j.it.2016.01.004 26858199 PMC4775398

[B23] Lauret Marie Joseph E. Laheurte C. Jary M. Boullerot L. Asgarov K. Gravelin E. (2020). Immunoregulation and clinical implications of ANGPT2/TIE2(^+^) M-MDSC signature in non-small cell lung cancer. Cancer Immunol. Res. 8 (2), 268–279. 10.1158/2326-6066.CIR-19-0326 31871121

[B24] Li L. Zhang H. Y. Zhong X. Q. Lu Y. Wei J. Li L. (2020). PSORI-CM02 formula alleviates imiquimod-induced psoriasis via affecting macrophage infiltration and polarization. Life Sci. 243, 117231 10.1016/j.lfs.2019.117231 31887296

[B25] Lu C. J. Yu J. J. Deng J. W. (2012). Disease-syndrome combination clinical study of psoriasis: present status, advantages, and prospects. Chin. J. Integr. Med, 18 (3), 166–171. 10.1007/s11655-012-1006-1 22466939

[B26] Lu C. Liu H. Jin X. Chen Y. Liang C. L. Qiu F., (2018). Herbal components of a novel formula PSORI-CM02 interdependently suppress allograft rejection and induce CD8^+^CD122^+^PD-1^+^ regulatory T cells. Front. Pharmacol 9, 88 10.3389/fphar.2018.00088 29483872 PMC5816027

[B27] May B. H. Zhang A. L. Zhou W. Lu C. J. Deng S. Xue C. C. (2012). Oral herbal medicines for psoriasis: a review of clinical studies. Chin. J. Integr. Med. 18 (3), 172–178. 10.1007/s11655-012-1008-z 22466940

[B28] Morgan E. Varro R. Sepulveda H. Ember J. A. Apgar J. Wilson J. (2004). Cytometric bead array: a multiplexed assay platform with applications in various areas of biology. Clin. Immunol. 110 (3), 252–266. 10.1016/j.clim.2003.11.017 15047203

[B29] Ogawa E. Sato Y. Minagawa A. Okuyama R. (2018). Pathogenesis of psoriasis and development of treatment. J. Dermatol. 45 (3), 264–272. 10.1111/1346-8138.14139 29226422

[B30] Ost M. Singh A. Peschel A. Mehling R. Rieber N. Hartl D. (2016). Myeloid-derived suppressor cells in bacterial infections. Front. Cell. Infect. Microbiol. 6, 37 10.3389/fcimb.2016.00037 27066459 PMC4814452

[B31] Parisi R. Symmons D. P. Griffiths C. E. Ashcroft D. M. Identification and Management of Psoriasis and Associated ComorbidiTy (IMPACT) Project Team (2013). Identification, Management of, Global epidemiology of psoriasis: a systematic review of incidence and prevalence. J. Invest. Dermatol 133 (2), 377–385. 10.1038/jid.2012.339 23014338

[B32] Soler D. C. Young A. B. Fiessinger L. Galimberti F. Debanne S. Groft S. (2016). Increased, but functionally impaired, CD14(+) HLA-DR(−/low) myeloid-derived suppressor cells in psoriasis: a mechanism of dysregulated T cells. J. Invest. Dermatol. 136 (4), 798–808. 10.1016/j.jid.2015.12.036 26807516

[B33] Sun S. Wei Y. Zeng X. Yuan Y. Wang N. An C. (2020). Circulating CD14(+)HLA-DR(−/low) myeloid-derived suppressor cells as potential biomarkers for the identification of psoriasis TCM blood-heat syndrome and blood-stasis syndrome. Evid. Based Complement Alternat. Med. 2020, 4582459 10.1155/2020/4582459 32382290 PMC7180989

[B34] Tsiganov E. N. Verbina E. M. Radaeva T. V. Sosunov V. V. Kosmiadi G. A. Nikitina I. Y. (2014). Gr-1dimCD11b+ immature myeloid-derived suppressor cells but not neutrophils are markers of lethal tuberculosis infection in mice. J. Immunol. 192 (10), 4718–4727. 10.4049/jimmunol.1301365 24711621 PMC4537794

[B35] Umansky V. Blattner C. Fleming V. Hu X. Gebhardt C. Altevogt P. (2017). Myeloid-derived suppressor cells and tumor escape from immune surveillance. Semin. Immunopathol. 39 (3), 295–305. 10.1007/s00281-016-0597-6 27787613

[B36] van der Fits L. Mourits S. Voerman J. S. Kant M. Boon L. Laman J. D. (2009). Imiquimod-induced psoriasis-like skin inflammation in mice is mediated via the IL-23/IL-17 axis. J. Immunol. 182 (9), 5836–5845. 10.4049/jimmunol.0802999 19380832

[B37] Wu D. H. Zhang M. M. Li N. Li X. Cai Q. W. Yu W. L. (2019). PSORI-CM02 alleviates IMQ-induced mouse dermatitis via differentially regulating pro- and anti-inflammatory cytokines targeting of Th2 specific transcript factor GATA3. Biomed. Pharmacother. 110, 265–274. 10.1016/j.biopha.2018.11.092 30513504

[B45] Wu H. Zhen Y. Ma Z. Li H. Yu J. Xu Z. G. (2016). Arginase-1-dependent promotion of TH17 differentiation and disease progression by MDSCs in systemic lupus erythematosus. Sci. Transl. Med. 23; 8 (331), 331ra40. 10.1126/scitranslmed.aae0482 PMC4895207 27009269

[B38] Wu S. Y. Chiang C. S. (2019). Distinct role of CD11b(+)Ly6G(−)Ly6C(−) myeloid-derived cells on the progression of the primary tumor and therapy-associated recurrent brain tumor. Cells. 9 (1), 51 10.3390/cells9010051 PMC7016541 31878276

[B39] Yue L. Ailin W. Jinwei Z. Leng L. Jianan W. Li L. (2019). PSORI-CM02 ameliorates psoriasis *in vivo* and *in vitro* by inducing autophagy via inhibition of the PI3K/Akt/mTOR pathway. Phytomedicine. 64, 153054 10.1016/j.phymed.2019.153054 31401494

[B40] Zhang C. S. Yu J. J. Parker S. Zhang A. L. May B. Lu C. (2014). Oral Chinese herbal medicine combined with pharmacotherapy for psoriasis vulgaris: a systematic review. Int. J. Dermatol. 53 (11), 1305–1318. 10.1111/ijd.12607 25208594

[B41] Zheng Y. Danilenko D. M. Valdez P. Kasman I. Eastham-Anderson J. Wu J. (2007). Interleukin-22, a T(H)17 cytokine, mediates IL-23-induced dermal inflammation and acanthosis. Nature. 445, 648 10.1038/nature05505 17187052

