# Supplementary material for: Chinese Medicine Formula PSORI-CM02 Alleviates Psoriatic Dermatitis via M-MDSCs and Th17 Crosstalk
Source: Front Pharmacol. 2021 Jan 18;11:563433. doi: 10.3389/fphar.2020.563433 (PMC7847847; doi:10.3389/fphar.2020.563433)
Supplement: Supplementary file 1 [file table1.docx]

**Supplementary Tab. 1 Chemical profiles of PSORI-CM02 formula (**[**Wu et al., 2019**](#_ENREF_34)**).**

| **NUM.** | **Retention time(RT, min)** | **Compounds** |
| --- | --- | --- |
| **1** | 3.0 | Gallic acid [a](https://www-sciencedirect-com.proxy.library.uu.nl/science/article/pii/S0753332218361699#tblfn0005) |
| **2** | 3.5 | 5-Hydroxymethylfurfural [b](https://www-sciencedirect-com.proxy.library.uu.nl/science/article/pii/S0753332218361699#tblfn0010) |
| **3** | 12.9 | Cryptochlorogenic acid [b](https://www-sciencedirect-com.proxy.library.uu.nl/science/article/pii/S0753332218361699#tblfn0010), [c](https://www-sciencedirect-com.proxy.library.uu.nl/science/article/pii/S0753332218361699#tblfn0015) |
| **4** | 15.6 | Albiflorin [d](https://www-sciencedirect-com.proxy.library.uu.nl/science/article/pii/S0753332218361699#tblfn0020) |
| **5** | 19.7 | Paeoniflorin [d](https://www-sciencedirect-com.proxy.library.uu.nl/science/article/pii/S0753332218361699#tblfn0020) |
| **6** | 21.1 | 5-O-caffeoylshikimic acid [a](https://www-sciencedirect-com.proxy.library.uu.nl/science/article/pii/S0753332218361699#tblfn0005), [c](https://www-sciencedirect-com.proxy.library.uu.nl/science/article/pii/S0753332218361699#tblfn0015) |
| **7** | 32.0 | Neoastilbin [a](https://www-sciencedirect-com.proxy.library.uu.nl/science/article/pii/S0753332218361699#tblfn0005), [c](https://www-sciencedirect-com.proxy.library.uu.nl/science/article/pii/S0753332218361699#tblfn0015) |
| **8** | 34.0 | Astilbin [a](https://www-sciencedirect-com.proxy.library.uu.nl/science/article/pii/S0753332218361699#tblfn0005), [c](https://www-sciencedirect-com.proxy.library.uu.nl/science/article/pii/S0753332218361699#tblfn0015) |
| **9** | 36.6 | Neoisoastilbin [a](https://www-sciencedirect-com.proxy.library.uu.nl/science/article/pii/S0753332218361699#tblfn0005), [c](https://www-sciencedirect-com.proxy.library.uu.nl/science/article/pii/S0753332218361699#tblfn0015) |
| **10** | 38.0 | Isoastilbin [a](https://www-sciencedirect-com.proxy.library.uu.nl/science/article/pii/S0753332218361699#tblfn0005), [c](https://www-sciencedirect-com.proxy.library.uu.nl/science/article/pii/S0753332218361699#tblfn0015) |
| **11** | 41.7 | Rosmarinic acid [c](https://www-sciencedirect-com.proxy.library.uu.nl/science/article/pii/S0753332218361699#tblfn0015) |
| **12** | 54.4 | 1-(3,4-dihydroxyphenyl)-5-hydroxy-7-(4-hydroxyphenyl)heptan-3-yl acetate [e](https://www-sciencedirect-com.proxy.library.uu.nl/science/article/pii/S0753332218361699#tblfn0025) |
| **13** | 56.7 | 1-(3,4-dihydroxyphenyl)-7-(4-hydroxyphenyl)heptane-3,5-diyl diacetate [e](https://www-sciencedirect-com.proxy.library.uu.nl/science/article/pii/S0753332218361699#tblfn0025) |
| **14** | 57.6 | Zederone [e](https://www-sciencedirect-com.proxy.library.uu.nl/science/article/pii/S0753332218361699#tblfn0025) |

a. Smilax glabra Roxb.

b. Prunus mume (Sieh.)Sieb. et Zucc.

c. Sarcandra glabra (Thunb.)Nakai.

d. radix of Paeonia lactiflora Pall.

e. radix of Curcuma phaeocaulis Val.
