# Supplementary figures and images for: Chinese Medicine Formula PSORI-CM02 Alleviates Psoriatic Dermatitis via M-MDSCs and Th17 Crosstalk
Source: Front Pharmacol. 2021 Jan 18;11:563433. doi: 10.3389/fphar.2020.563433 (PMC7847847; doi:10.3389/fphar.2020.563433)

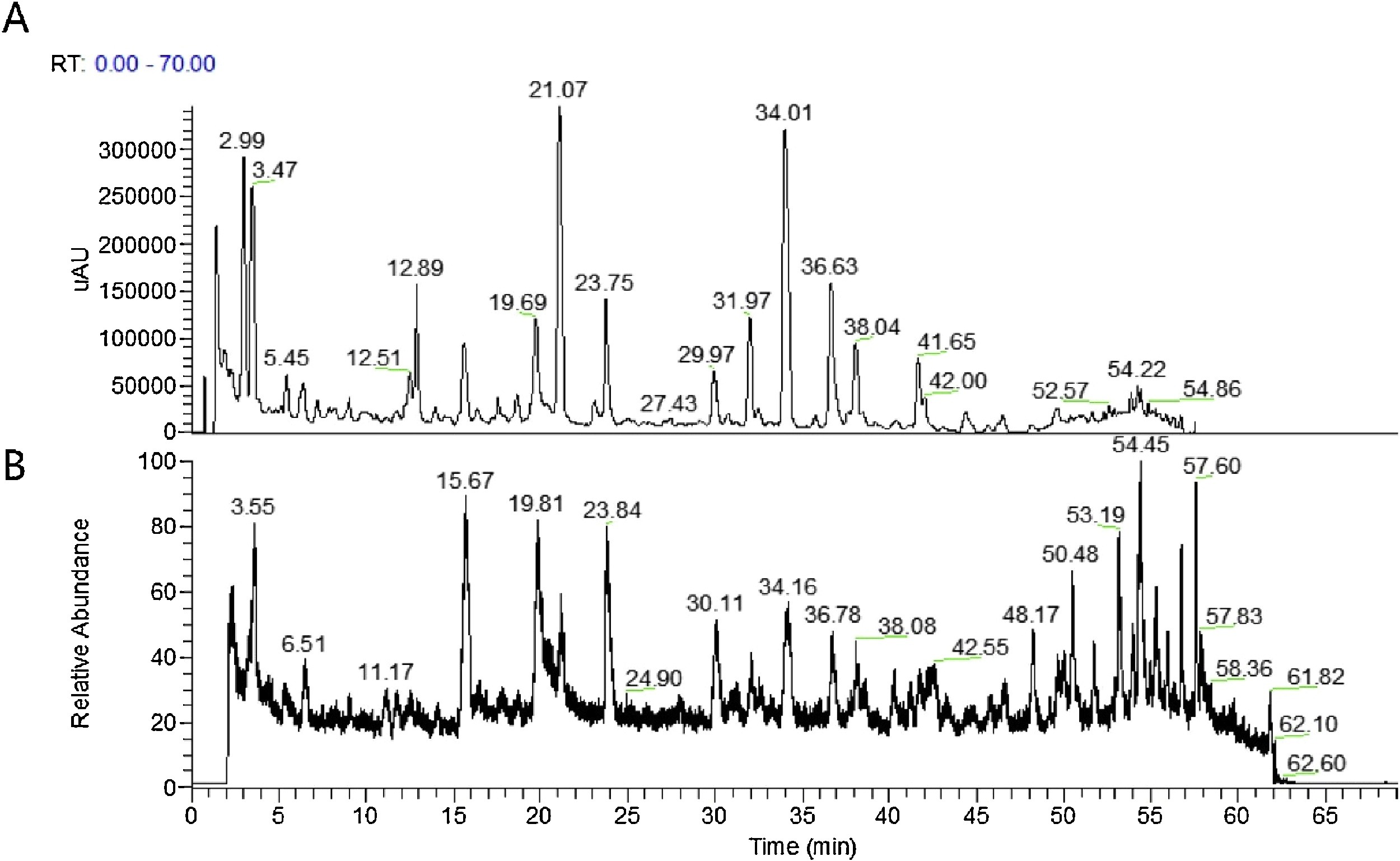

Supplement: Supplementary file 2 [file image1.jpeg]
